# Cold Water Dangerous

**Published:** 1872-11

**Authors:** 


### COLD WATER DANGEROUS.

Like any other blessing, cold water has its victims, and in one way or other, they are numerous every year; more now than ever before, since the cold water bathing mania has taken complete possession of a certain class of minds, not of the highest grade.

A distinguished commodore of our navy, the son of an illustrious father, died within a month, at but an hour's notice, from taking a cold bath in August.

The more intelligent and observant of the water-cure men have already found it necessary to give repeated admonitions in their journals of health, and caution their adherents against the indiscriminate use of cold water, even for drinking purposes. Dr. Trall has just come to the front in this connection, and the influence of his name and position will cause a "pause" in the fanatical admiration of this, one of the best gifts to man.

Multitudes of the more unreasoning are completely "possessed" with the idea that, as water is pure and cleansing, the larger a stream of it a man can put through himself, the healthier and purer and cleaner he will be; hence, not a few drink quarts of it a day, without having any thirst for it whatever. Many others are carried away with the delusion that a glass or two, or more, of fresh water drank in the morning, soon after rising,

IS SPLENDID; that it washes out the mouth, stomach, intestines, and all there is left in the brains! Dr. T. has attacked this misapprehension also, by presenting this most convincing idea. All the water drank must be worked out of the system, every drop of it, by appropriate vessels; that healthy persons seldom have any special thirst except after overwork or overeating, and that if only a pint a day is thirsted for, and a gallon is drank, not thirsted for, then seven times more work is imposed upon the delicate organs of the system than is really necessary, and that such extra work, long continued, must inevitably induce disease. It is just as unphilosophical and unphysiological to drink a drop of water when you are not thirsty, as it is to eat a particle of food when there is no hunger for it. The familiar saying is well worthy of repetition now and then, "Eat when you are hungry, drink when you are dry," with the important addition, "and only then." It cannot be doubted for a moment, that a judicious attention to this plain and clear and truthful admonition, would prevent many an attack of illness, would forestall many an agony, would save many a person from a premature grave. To eat when one is not hungry, as a necessity for keeping up the strength, is one of the veriest absurdities of the time; but an absurdity that finds adopters in almost every uncultivated family, and even among some who ought to know better from instinct, — as even the brutes do!

Very lately a mother, while unwell, put her feet in cold water, and died in consequence of the same, in a very few weeks.

Washing the feet in cold water, while the body is overheated, has repeatedly cost serious illness, of weary weeks' duration.

The only son of a wealthy New Yorker, while camping out during the war, went to sleep one summer's night with his feet in a running rivulet, and died of inflammation of the lungs within six days. There is scarcely a single remedial effect of cold water, which cannot be as well and more safely induced by warm water, in one way or another. Never use cold water, when warm will answer as well.

